# Plant Movement Response to Environmental Mechanical Stimulation Toward Understanding Predator Defense

**DOI:** 10.1002/advs.202404578

**Published:** 2024-08-29

**Authors:** Alex Naglich, Philip LeDuc

**Affiliations:** ^1^ Department of Mechanical Engineering Carnegie Mellon University Pittsburgh PA 15213 USA; ^2^ Department of Biological Sciences Carnegie Mellon University Pittsburgh PA 15213 USA; ^3^ Department of Biomedical Engineering Carnegie Mellon University Pittsburgh PA 15213 USA; ^4^ Department of Computation Biology Engineering Carnegie Mellon University Pittsburgh PA 15213 USA; ^5^ Department of Electrical and Computer Engineering Carnegie Mellon University Pittsburgh PA 15213 USA

**Keywords:** biohybrid systems, mechanics, mimosa pudica, plants, signal response

## Abstract

Plants are fascinating living systems, possessing starkly different morphology to mammals, yet they have still evolved ways to defend themselves, consume prey, communicate, and in the case of plants like *Mimosa pudica* even move in response to a variety of stimuli. The complex physiological pathways driving this are of great interest, though many questions remain. In this work, a known responsive plant, *M. pudica* is mechanically stimulated, in terms of wounding via removal of pinnae, nonwounding mechanical poking, and nonwounding pulses of air through a designed small nozzle approach. Removal of clusters called pinnae resulted in rapid, asymmetric response in the adjacent pinnae, while mechanical poking and air pulse responses are slower and more localized. Additionally, while the response from poking propagated across the plant, wind stimuli consistently resulted in the actuation of only the leaflets directly stimulated, suggesting unique sensing mechanisms. Mechanical damage may imply a potential predator, while mechanical stimulation from airflow may be processed as wind, which is of little danger. These findings demonstrate an intricate, stimulant‐dependent mechanical sensing process, which is important in plant physiology, mechanobiology, and future biohybrid soft robotic designs.

## Graphical abstract

While morphologically distinct from animals, plants evolved complex evolutionary solutions – including *M. pudica's* rapid mechanical motion. Wounding (predator emulation) produces rapid, asymmetric responses in surrounding organs while poking (falling leaves) and air pulse (wind) responses are slower and localized. Additionally, poking responses propagate while wind does not. This suggests an evolutionary advantage to developing individualized responses for distinct mechanical stimuli.

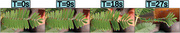

## 1 Introduction

Plants play a vital role in the perseverance of life on Earth. From individual cells to the largest and oldest organisms in the world, the shelter, food, and nutrient exchange provided by the wide variety of plants is a keystone of ecosystems everywhere. Examples of their importance to life are that plant sugars produced through photosynthesis provide the energy for most ecosystems, terrestrial and marine plants produce most of the planet's oxygen, and 80% of Earth's biomass is from plants^[^ ^1^ ^]^ Plants come in a stunning array of forms and possess countless features that have led to their widespread success. One such feature is large‐scale movements, typically associated with animals. Most plants are sessile though and cannot make macroscopic movements^[^ ^1^ ^]^ Slower movements, such as bending to track the direction of sunlight (phototropism)^[^ ^2^ ^]^ or the cyclic folding of leaflets with response to the daily light and temperature cycle (nyctinasty)^[^ ^3^, ^4^ ^]^ are common and widely seen in plants. Many of these movements are well described. Additionally, plants use other types of sensing to respond to stimuli including electric signals, which send local information, and signaling such as that in *Arabidopsis thaliana* are also well studied and understood^[^ ^5^ ^]^ However, observable, rapid movement—such as that in the carnivorous Venus flytrap and explosive bunchberry dogwood—are less frequently encountered in nature,^[^ ^6^, ^7^ ^]^ and efforts to understand the nature of their underlying mechanics and signaling pathways continue.

**Figure 1:**
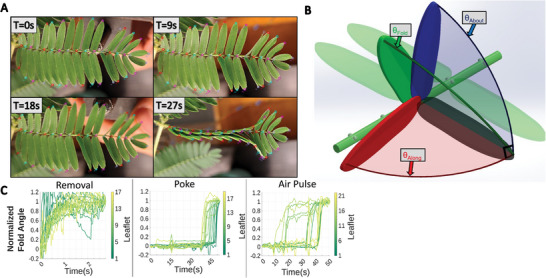
A) Four snapshots of *M. pudica* during stimulation via poking with blunt AWG23 (American Wire Guage) metal tip, showing a propagation of actuation between T = 18 s and T = 27 s. B) Kinematic coordinate system used. θ_about_(blue) is the angle rotated around the rachis. θ_along_(red) is the angle rotated along the pinna toward the end of the rachis. θ_fold_(green) is the combined angle about both axes. A diagram of plant characteristics is in Figure 2. C) Comparison of the response of selected examples from each of the three modes of stimulation (removal, poke, air pulse) showing the dramatically different shape of the behaviors as well as the average delay between one leaflet achieving 20% normalized actuation and the next leaflet and standard deviation. Each line is a leaflet, with time on the X‐axis and the progression of folding represented on the Y‐axis. Line brightness shows proximity of leaflets to their neighbors. Additional details are presented in Figure 3, 4, 5, and photos demonstrating experimental setup available in Figure S5–S7 (Supporting Information).

**Figure 2:**
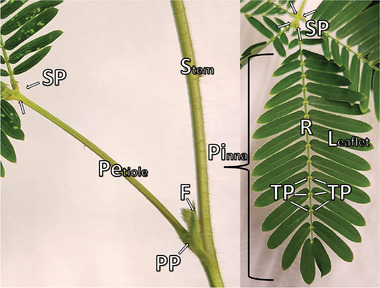
Photographs of the considered parts and organs of the *Mimosa pudica*; left: lateral view, right: top view. The primary (PP), secondary (SP), and tertiary (TP) pulvini are the motor organs of the *Mimosa pudica*, moving the Petiole (P), Rachis (R), and Leaflet/Pinnule (L) respectively. Also pictured are the Stem (S) and Flower (F).

*Mimosa pudica* responds to a variety of stimuli with the rapid movement of various organs across its structure, as seen in **Figure** **1**.^[^ ^8^ ^]^ This motion is provided by motor organs (i.e., pulvini in **Figure** **2**) and the most dramatic of these responses is the rapid closure of the leaflets in the pinna when exposed to mechanical touch (known as thigmonasty), flame, damage, and other stimuli.^[^ ^9^ ^]^ This response has been theorized to be an evolutionary advantage to protect against predators by exposing thorns^[^ ^10^ ^]^ or to present a seemingly less voluminous meal^[^ ^11^ ^]^ and recent work by Hagihara et. al. has demonstrated these movements protect from insect attacks.^[^ ^12^ ^]^ A response will also be observed, however, in non‐damaging scenarios, such as acute touch and exposure to wind.

*M. pudica* is a tropical member of the legume family, native to the Caribbean and South/Central America. The plant's leaves are bipinnately compound, with 1–3 pinnae pairs appearing together and each pinnae having 1–2 dozen leaflets. To respond to so many different stimuli, and to do so with different signals, suggests an evolutionary advantage to this complexity‐ or, at minimum, negligible costs to survival. Having different signaling pathways to carry responses to different signals would allow the same actuation system to cater the response proportionally to the threat the plant is experiencing. A predator wounding the plant, for instance, would warrant a more dramatic response than a light wind. Stimulating and observing *M. pudica* under various mechanical stimuli meant to emulate stimuli experienced by *M. pudica* in nature may reveal unique macroscopic behaviors and inform our understanding of what evolutionary advantages these responses afford to the plant that justifies the development of such unique sensors and pathways.

Turgor pressure changes in the pulvini drive this motion. Cells in the pulvinus will either grow (flexor cells) or shrink (extensor cells), and these volumetric changes move the plant leaves, but it has been reported that motion in the primary pulvinus can occur with only the extensor cells.^[^ ^13^ ^]^ While there is evidence of association between turgor changes and efflux of K^+^ and Cl^+^ ions from cells,^[^ ^14^, ^15^, ^16^ ^]^ which has been confirmed as a fundamental precursor to turgor loss^[^ ^17^ ^]^ the aquaporin protein's effect on water flux^[^ ^18^ ^]^ and the migration of colloidal substance in the pulvini^[^ ^19^ ^]^ are also key factors. The mechanisms of turgor change in *M. pudica* are still being explored. Similarly, there are gaps in understanding the triggering and signaling of this response. The behavior varies depending on the source of stimulus, such as non‐wounding (touch, shock, electricity) or wounding (cutting or burning).^[^ ^20^, ^21^ ^]^ Additionally, folding can occur in response to nyctinastic stimuli, which uses different motor cell groups from mechanical stimuli^[^ ^22^ ^]^ and even a transition to darkness can trigger folding.^[^ ^23^, ^24^ ^]^ Mechanosensitive ion channels have been identified that relate to folding behavior^[^ ^25^ ^]^ but there is evidence for additional conduction methods for mechanic stimuli^[^ ^26^ ^]^ Signaling mechanisms have been defined by Houwink into three major categories:^[^ ^21^ ^]^ The action potential, which moves quickly but does not propagate through the system independently.^[^ ^21^, ^27^, ^28^ ^]^ The variation potential, a slower wound‐triggered signal that propagates and enables the action potential and is thought to be linked with chemical stimuli that may travel through the xylem.^[^ ^9^, ^21^, ^28^, ^29^ ^]^ Finally, an “unknown rapid” third signal, the fastest signal—produced by major wounding and can cause actuation before the arrival of the other signals, perhaps by hydraulic action.^[^ ^8^, ^21^, ^26^ ^]^ There is limited work into *M. pudica's* behavioral response to wind pressure stimuli. Kimata et. al observed the signal waveforms generated by different wind pressures and identified four waveform types.^[^ ^30^ ^]^

Exploring new actuators for biohybrid soft robot designs using plants presents an exciting opportunity. Soft robots are predicted to lead to the development of machines with interesting abilities, such as climbing, growing, squeezing, and morphing. Biological actuators capitalize on the progress and efficiency of millions of years of evolution to meet these goals.^[^ ^31^ ^]^ Designs thus far have created interesting applications such as insect cyborgs, remote‐controlled jellyfish, inchworm‐styled robots using sea‐slug muscle, and more.^[^ ^32^ ^]^ However, few designs have utilized plants, with a notable example being a robotic gripper developed by Li et al. that triggered venus flytrap actuation on command to pick up a wire.^[^ ^33^ ^]^ With this said, plants hold much promise for future developments – as discussed by Kumar et al. plants are highly complex adaptable species that are able to discriminate between positive and negative experiences, adapting and “learning” from these experiences – potentially demonstrating a form of primitive cognition.^[^ ^34^ ^]^ Understanding these evolutionary adaptations and behaviors emerging from this primitive cognition could pave the way for plant‐based biohybrid designs with integrated logic in the form of the actuating body itself.

Amongst such “intelligent” plants, *Mimosa pudica* presents a promising actuation candidate for soft biohybrid designs. The primary pulvinus provides 6.95Nm kg^−1^ torque/mass and 6.95 W kg^−1^ power/mass, with an absolute torque output ≈50mNmm. It is similar for power storage to shape memory alloy, and the smaller pulvini have diameters as small as 0.2 mm.^[^ ^35^ ^]^ On top of this impressive power density, small size, and high efficiency *M. pudica* pulvini have some survivability after being removed from the main body and are fully biodegradable. Aishan et al. take advantage of these features to use a cut sample of *M. pudica* to act as a sensor/actuator pair for a microfluidic valve. Testing with uncut samples produced a force of ≈16mN that could open the valve for ≈8 min, and using two pulvini from a cut sample could open the valve for ≈2 min.^[^ ^36^ ^]^ *Mimosa pudica* has also been documented to exhibit many examples of complex learning and plant intelligence, such as habituation and stimuli discrimination.^[^ ^37^ ^]^ Understanding the sensing, signaling, and behavior of *M. pudica* will provide useful insight for future integration of its pulvini into such soft robotic designs.

In this work, we compare the behavioral differences between wounding (active predation), nonwounding solid (potential predation), and nonwounding wind pressure (environmental wind) mechanical stimuli by tracking the movements of individual leaflets building off our previous work in plant physiology^[^ ^38^ ^]^ and mechanotransduction.^[^ ^39^, ^40^ ^]^ We expose plants of *M. pudica* to each of these stimuli and record their response. By tracking folding of leaflets and using a quantitative model developed for this experiment, the mechanical response across leaflets is recorded and analyzed for each stimulus. This work may provide potential insights into the plant's structure and mechanics and clues for explaining the sensing and signaling of *M. pudica*. The useful understanding of *M. pudica*’s kinematic behaviors also may inspire biohybrid systems.

## 2 Results

### 2.1 Predation Inspired Mechanical Stimulation: Pinna Removal

**Figure 3:**
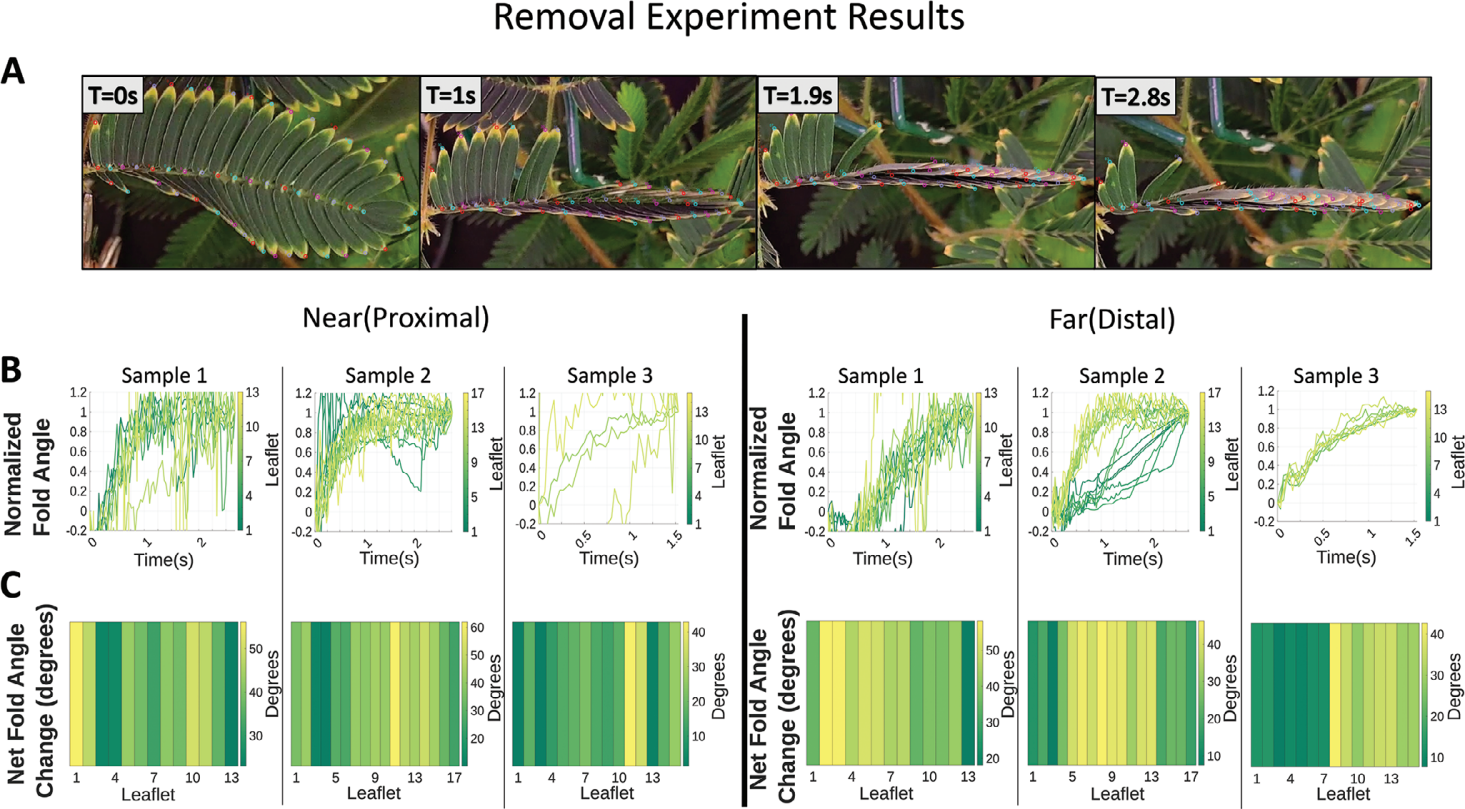
*Mimosa pudica* folding response to wounding stimulation via removal of adjacent pinna by means of cutting with scissors, separated into the halves of pinnae divided by the rachis that are proximal(near) and distal(far) to the removed pinnae. A) Four snapshots of an example cutting of a sample of *M. pudica*, showing the frame immediately following the initial cut, T = 0 s. The reaction occurred very quickly, with most folding complete by T = 1 s, and only small additional changes between T = 1 s and T = 2.8 s. B) Line graphs show the folding of leaflets with respect to time. Time = 0 corresponds to the application of the first stimulus. The Y‐axis has normalized to express the initial folding as “0” and the final folding as “1”, with unresponsive leaflets removed for clarity. Line graphs show rapid, mostly simultaneous response in both sides, with leaflets on the distal half of the rachis of the removed pinna (labeled “Far”) having a slower initial response than leaflets on the proximal half (labeled “Near”). Leaflet 1 is the basal leaflet, with each leaflet moving distally incrementing by 1. C) Heat maps show a total difference in angle between the least and greatest fold values (in degrees) observed in the observational period. The heat maps show that mostly all leaflets responded in samples 1 and 2, with no clear pattern emerging. However, in sample 3 only the leaflets further from the base of the pinna with higher numbers responded, meaning the signal traveled through the basal leaflets without triggering their actuation. Photos demonstrating the experimental setup are in Figure S5 (Supporting Information).

Understanding how the plant responds to wounding stimuli—sensing and signaling even upon destruction—will aid in understanding how plants survive amid dangers such as predation and the evolutionary mechanisms they employ. Our first experiments focused on mechanical stimulation that was related to cutting of the plant. For this test, a pinna was removed completely using a small pair of scissors positioned at the base of the rachis, and observation was done on the pinna adjacent to the one removed. Removal resulted in an immediate chain reaction in the adjacent pinna, as seen in **Figure** **3**. In seconds (<3s) most/all leaflets on a pinna fully fold. This is consistent with observed reaction times, which are proportional to the intensity of the stimulus.^[^ ^21^, ^29^ ^]^ This data shows the response of the leaflet rows on the near and far side of the removed pinnae, where “near” corresponds to the leaflets on the proximal half of the rachis, and “far” the distal half. The far row in samples 1/2 had a slower mean time to 20% actuation (1.1/0.63 s) than the near rows (0.43/,15 s), while times were similar across sample 3 (0.1/0.7 s). This asymmetric response may suggest that each side has independent signalling pathways, supporting Bose's findings that signals in *M. pudica* follow two distinct strands along the pinna through the central rachis.^[^ ^41^ ^]^ The faster response on the near side of the observed pinna may also suggest that these “strands”, or independent signal pathways, are more strongly linked between the adjacent halves of the pinnae. Interestingly, in all three “far” trials the first actuation occurred in the basal leaflets closer to the base of the pinna, followed by the distal leaflets further from the base of the pinna (in sample 3, only the distal leaflets actuated on both sides). This suggests the signal went through the basal leaflets without actuating to reach the proximal leaflets. This resembles the “unknown” rapid movement^[^ ^21^ ^]^ as the signal precedes the action and variation potentials that would have traveled acropetally, or base to tip. The larger movement produced by the folding of not only the affected pinna but also the pinnae adjacent to those damaged may serve to scare off larger predators who would otherwise be unphased by the small, local movements observed in nonwounding scenarios.

### 2.2 Predation Inspired Mechanical Stimulation: Acute Mechanical Touch Stimulation

**Figure 4:**
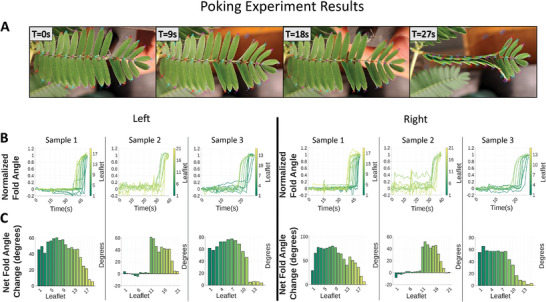
*Mimosa pudica* folding response to stimulation on back of leaflets via mechanical prodding with blunt AWG23 metal tip. A) Four snapshots of an example poking of a leaf of *M. pudica*. Frames show the progression of folding with subsequent pokes between T = 0 s and T = 18 s, followed by a cascade of actuation between T = 18 s and T = 27 s. B) Line graphs show the folding of leaflets with respect to time. Time = 0 corresponds to the application of the first stimulus. The Y‐axis has normalized to express the initial folding as “0” and the final folding as “1”, with unresponsive leaflets removed for clarity. Line graphs show organized, regular folding in leaflets, in contrast with air pulse experiments (Figure 5). Leaflet 1 is the basal leaflet, with each leaflet moving distally incrementing by 1. C) Bar charts show the total difference in angle between the least and greatest fold values observed in the observational period. Bar charts show that the signal in sample 2 started near the middle and moved toward the end, and in samples 1 and 3 signal started at the ends and moved toward the base. This propagating, directional behavior suggests a variation potential was produced by non‐wounding stimuli. Photos demonstrating experimental setup available in Figure S5 (Supporting Information).

Touch is the stimulus that *M. pudica* is most known for, and this will provide a baseline for comparison against the other experiments as well as existing work to understand the differences in mechanosensory response of these unique plants. Thus, we next focused on non‐wounding mechanical stimulation from poking with a blunt metal tip. A technique was designed to move the metal tip into the rear of the leaflets at a linear velocity. This mechanism was mounted to the end of a stiff but orientable arm, allowing positioning of the tip without disturbing plants. Once positioned, leaflets were poked until a “propagation wave” was observed. Initially, only the directly stimulated leaflets would fold—if only slightly—upon being poked, but following repeated stimuli (three in samples 1 and 2, six in sample 3) a cascade of actuation is seen moving across the plant, folding each leaflet in succession (**Figure** **4**). The initial localized, non‐propagating behavior resembles the action potential, which is observed to be unable to travel through the pulvinus.^[^ ^21^, ^27^, ^28^ ^]^ This stimulus was not wounding (blunt metal tip), yet propagation occurred. This is likely due to variation potentials, which suggests additional mechanisms for generating a variation potential beyond wounding. In addition, interestingly, signal propagation from the first point of actuation was basipetal in samples 1 and 3, and acropetal in sample 2. Signals propagated in only one direction.

### 2.3 Environmental Mechanical Stimulation/Wind: Air Pulse

**Figure 5:**
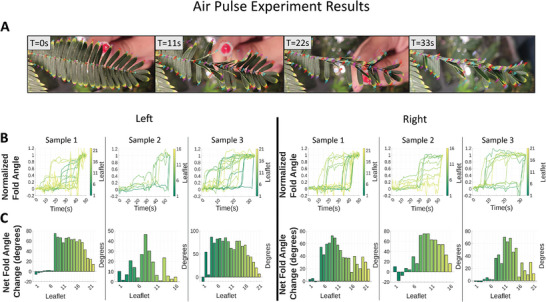
*Mimosa pudica* folding response to stimulation on back of leaflets via pulses of air at 6 kPa from a 25Ga nozzle for 50 ms by means of a small nozzle connected to a solenoid valve. A) Four snapshots of an example air pulse on a sample of *M. pudica*. Frames show numerous local, single leaflet responses between T = 0 s and T = 33 s, but no propagation through the pinna. B) Line graphs show the folding of leaflets with respect to time. Time = 0 corresponds to the application of first stimulus. The Y‐axis has normalized to express the initial folding as “0” and the final folding as “1”, with unresponsive leaflets removed for clarity. Line graphs show nonuniform folding in leaflets with individual leaflets actuating independently, in great contrast with poking experiments (Figure 4). C) Bar charts show the total difference in angle between least and greatest fold values observed in the observational period. Bar charts show total difference in angle between least and greatest fold values observed in the observational period, and show no distinct points where folding signals originated and moved in one direction, as in the poking experiments (Figure 4). Photos demonstrating the experimental setup are in Figure S7 (Supporting Information).

To enable comparison with the previous experiments where individual leaflets were poked, a pneumatic system was designed to independently simulate wind individual leaflets. A solenoid valve connected to an air compressor was opened for brief periods of time to allow pulses of air. This air was moved through a tube to a nozzle positioned behind the leaflet to be stimulated and passed through a small nozzle to ensure only one leaflet was stimulated. Upon stimulation, an initial behavior similar to the mechanical poking test is observed—folding of only individual stimulated leaflets (**Figure** **5**). However, in contrast to the mechanical poking tests, the wind pressure tests never produced a propagating wavefront of folding through the leaflets. Additionally, the initial movement here is much larger. Where the initial poking movements visible in Figure 4A are indiscernible in the graphs in Figure 4B,C, they are distinct in the air pulse graphs here in Figure 5B,C.

The fold angle data (Figure 5B,C) is also quite different from the mechanical poking (Figure 4B,C). The poking tests show a noticeable point where the pulvini of multiple leaflets actuate in close temporal proximity, while the air tests are completely individual and do not appear to show any clear grouping.

## 3 Discussion

**Figure 6:**
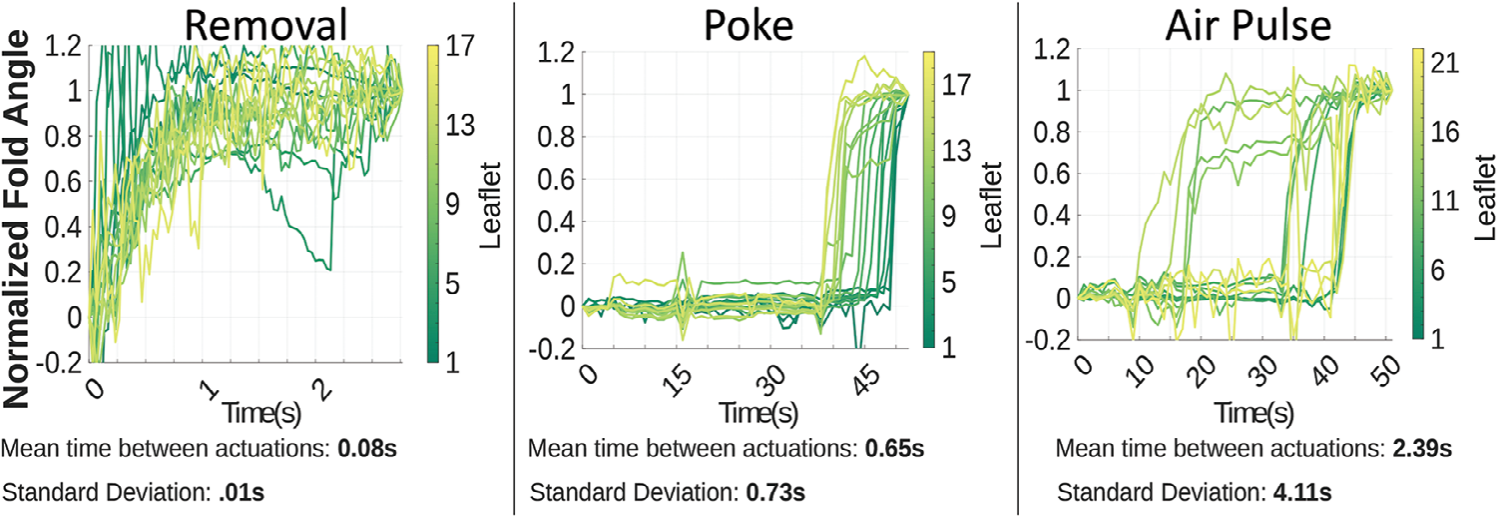
Comparison of the response of selected examples from each of the three modes of stimulation (removal, poke, air pulse) showing the dramatically different shape of the behaviors as well as the average delay between one *Mimosa pudica* leaflet achieving 20% normalized actuation and the next leaflet and standard deviation. These values are approximately an order of magnitude different between each experiment moving from A) Wounding experiments where a pinna, or leaf cluster, is removed and the adjacent pinna observed, B) non‐wounding prodding of the back of leaflets using a blunt metal tip and C) non‐wounding pulses of air directed through a small nozzle onto the back of the leaflets.

The responses observed in the three testing scenarios clearly demonstrate unique responses for each of the mechanical stimulations and suggest an evolutionary incentive guiding development of this ability to respond to mechanical threats. The results of the poking and air pulse tests, which exhibit dramatically different behaviors (**Figure** **6**), suggest unique triggering mechanisms for the stimuli. If mechanosensitive cells provide the initial signal for pokes/touch^[^ ^25^ ^]^ another cell may have specialized for wind stimuli. Alternatively, the response may be more structural, detecting or resulting from deformation at the pulvinus or in the cell walls. The signal resulting from this different sensor provides a different signal from that of the mechanosensitive cells, one that does not create a variation potential or requires much more intensive stimulus. It is also possible that this behavior is the result of the wind placing small enough stresses on the mechanosensitive cells to create an action potential, but not a variation potential.

An interesting connection may also be found between our findings and the three‐part model provided by Houwink that identified the action potential, variation potential, and an unknown third signal pathway.^[^ ^21^ ^]^ The localized behavior followed by a propagating signal in the poking experiments both supports the observation of the action potential being unable to bypass pulvini^[^ ^21^, ^27^, ^28^ ^]^ as well as the variation potential being able to override/bypass this.^[^ ^28^ ^]^ The pinna removal experiment demonstrates the rapid response of the unknown third signal pathway, as well as supporting the third pathway's ability to bypass pulvini and send long‐distance signals. The air pulse experiments clearly demonstrate the kinematic differences between a localized and a propagating response and support models that distinguish between the two behaviors, such as Houwink's.

Additionally, the removal experiment reinforces Bose's two‐stand signal model^[^ ^41^ ^]^ while also suggesting that there is additional interaction between the proximate strands on adjacent pinnae. The poking experiment, if demonstrating the variation potential, suggests that propagation of the variation potential may be triggered by more than just wounding. Variation potential generation could occur from unique sensors, or from reaching a critical threshold of signal or concentration from proximate leaflets—perhaps by buildup of the chemical signals currently suggested to travel through the xylem and mediate the variation potential.^[^ ^9^, ^21^, ^29^ ^]^ The air pulse experiments suggest the possibility of alternate sensors and/or signals for wind stimuli, but may also be the result of identified mechanosensitive channels being stimulated by the stresses resulting from the wind.^[^ ^25^ ^]^

The tip‐to‐base response in the removal tests is interesting. The behavior described where the end leaflets respond to removal first occurs on the “far” side of all three experiments, and the “near” side of only sample 3. Why this rapid behavior is preferential to the “far” row of pinnules required is unknown but may simply be due to the near‐simultaneous response of every leaflet on the two samples where this was not observed. Either way, further exploration in the future into the mechanisms converting this rapid, long‐distance signal into local actuation would provide key insights into the function and operation of this intriguing signaling pathway.

These results suggest an evolutionary benefit to having unique responses to different stimuli. The total response observed in the removal experiments makes the plant less visually concealing – accordingly, any herbivores attempting to predate *M. pudica* may potentially find themselves more exposed to predation themselves by nearby carnivores, now able to see their prey. Looking toward the poking experiments, the propagating response resembles descriptions of the variation potential, which can travel through pulvini and trigger action potentials.^[^ ^28^ ^]^ Wounding is typically cited as the difference between triggering solely an action potential and triggering both an action and variation potential.^[^ ^20^, ^21^ ^]^ Following the hypothesis that the variation potential is mediated by chemical signals,^[^ ^9^, ^21^ ^]^ it may suggest a trigger other than wounding for generating a variation potential. By folding the pinna directly affected by nonwounding stimuli, the plant may scare off smaller predators, like insects, while still allowing the rest of the leaflets (which did not fold) to continue to work on a primary function of theirs – photosynthesis. Finally, for the air pulse experiments, exposure to violent gusts of wind may damage plants, so decreasing the surface area of the leaflets by folding would protect them from damage. *M. pudica* may respond accordingly to wind stimuli. What is observed is a response only in the leaflets exposed to this wind stimuli. One possible explanation of this behavior could be that it provides an evolutionary advantage, allowing *M. pudica* to only close the parts of itself that are being exposed to fierce winds, as the parts that are not experiencing wind are not in danger unless the wind changes. This contrasts with wounding and solid touch stimulus, which may damage or touch the plant again.

## 4 Conclusion

Research into rapid movement plants continues to provide a window into the understanding of the development and structure of plant physiology. In this work, stimulated *M. pudica* via wounding cutting, non‐wounding poking, and non‐wounding wind simulation pulses were implemented through our controlled approaches designed to mimic threats faced by *M. pudica* in nature. With this, our goal is to start to observe how *M. pudica* has developed tailored thigmonastic response patterns to best respond to these threats for survival. Quantitative folding data was obtained by optically tracking leaflets and using a model to extract values for folding of each leaflet. This data provided insights into the behavior and mechanical response from these stimuli, such as observations of asymmetric responses across pinnae following wounding stimuli and distinct differences between mechanical poking and wind. Our results both provide support for existing signaling theories and suggest new complexities to existing behaviors and signaling pathways as well as how plants may integrate different stimuli into their responses.

Future efforts may have several interesting directions including exploring the development of *M. pudica*‐based biohybrid systems. Biohybrid systems show potential to provide devices with a high power/mass ratio, and soft, low‐wattage actuation.^[^ ^31^ ^]^ *Mimosa pudica* is a great candidate for this, as it is easier to grow and maintain than other biohybrid actuators. Additional work will further explore the relationship between adjacent pinnae to better understand the role structure plays in *M. pudica*'s survival. We are also interested in isolating the variation potential from the action potential to determine if generation of variation potential depends upon the action potential or if generation occurs via other mechanisms. This future work will provide a useful understanding of *M. pudica*’s kinematic behaviors inspiring biohybrid systems, provide clues for understanding the sensing and signaling of *M. pudica*, and provide potential insights into the development and structural mechanics of the plant kingdom.

## 5 Experimental Section

### Growing and Testing *Mimosa pudica*

*Mimosa pudica* seeds were sourced from Outside Pride(Amazon, USA). Seeds were soaked in DI water for 24 h, then placed at a depth of ⅛in. in Miracle‐Gro Moisture Control Potting Mix(Home Depot, USA). Germination was done using #1206 inserts placed in #1020 trays and covered in 7″ vented humidity domes from Greenhouse Megastore(Greenhouse Megastore, USA). Trays were kept flooded with a solution of 1tsp Maxsea brand plant food(Amazon, USA) per 1L DI water to ensure consistent water and micronutrient availability, as both may affect leaflet folding behavior. As plants sprouted and grew, the humidity dome was removed, and plants were transferred to progressively larger containers to allow room for root structures to grow. The light was provided by Monios brand T5 LED grow lights: white(Amazon, USA). The height of the light with respect to plants changed through the lifespan of plants to prevent overexposure, but multiple lights ensured all plants had sufficient light. The schedule was controlled using BN‐LINK BND‐60/U58 24h mechanical timer outlets(Amazon, USA). Plants were given 16h of light per 24h period. Dead leaves and stems were removed periodically with scissors. Support was occasionally provided to prop plants that were growing too low—this was accomplished using a variety of generic garden supports and twist ties. No change in behavior was observed following the application of support. No disease was observed during the course of the experiment. All experiments were done on healthy adult plants, 1.5–3 months old. The temperature of the air during testing was ≈80 F, the humidity was RH50%, and the light intensity was 8000–16 000 lux (depending on proximity to growing lights).

Tested leaflets were similar in age, being mature such that the leaflet tips were near to or exhibiting chlorotic tips. Leaflets of this age extended perpendicular to the rachis (while younger leaflets did not), which was necessary to prevent folding leaflets from occluding each other in camera capture. The behaviors documented in this paper were observed in all ages and all locations, but other ages were more difficult to capture data. Tests were conducted upon 9 different plants, with data acquired from 1 pinna from each of these nine plants appearing in the data shown in the above Results section. Each pinna was stimulated once to avoid fatigue.

### Pinna Removal

Pinnae were removed using a small pair of stainless‐steel scissors by hand. The cut was done at the base of the rachis near the secondary pulvinus. One of the two central pinnae was removed, and then the adjacent exterior pinnae, left isolated, was analyzed.

### Pinnule Poking

Poking was implemented (Figure S2, Supporting Information) through a Nema17 stepper motor connected to a custom designed cam to provide constant tip velocity for tests. This was mounted to the end of a Heavy‐Duty Aluminum Gooseneck Tablet Holder (AboveTEK, Amazon, USA) for stability. An AWG23 (American Wire Guage) jumper wire with a blunt end was used, secured into the end of the swappable tip holder using thermal responsive adhesive. Full assembly can be found in Figure S2 (Supporting Information). The tip was positioned ≈1 cm from the rear of the distal half of leaflet. Once positioned, the tip moved forward at a rate of 4.3 mm s^−1^ for ≈10 mm to poke the leaflet. After this, another unactuated leaflet was poked until a cascade response was observed.

These parameters were chosen to avoid damage to the plant (slow speed and blunt tip) and to avoid any mechanical vibrations (a displacement and velocity were selected that provided sufficient stimuli to induce folding but did not cause any additional “issues”, such as the leaflet slipping off the end effector or the speed of the poke causing vibrations to travel to other leaflets).

### Air Pulse

A 1/5HP Airbrush Compressor with Regulator (Pointzero, Amazon, USA), Pneumatic Solenoid Valve (TAILONZ PNEUMATIC, Amazon, USA), and Uno R3 Microcontroller (Arduino, Amazon, USA) were used to produce 50 ms duration pulses of air at 6 kPa through a 25Ga blunt‐tipped syringe positioned ≈1 cm from the rear of the distal half of leaflet. Leaflets were stimulated until most were fully folded.

The selected parameters (distance, pressure, nozzle diameter) were chosen to fit a few repeatable criteria: First, the wind did not (visibly) affect any of the other leaflets to ensure both experiments only stimulated one leaflet at once. Second, the displacement of the leaflet caused by the poking experiment and the airpulse experiment were similar. Finally, the nozzle diameter was small and close to the leaflet such that the area and intensity were similar.

### Video Analysis and Tracking

The video was captured using a Samsung A50 camera at 30fps, 1080p. For the removal test, the full 30fps was used, and for the slower poking and air tests, the video was reduced to 1fps using the HandBrake video editor's Command Line Interface(CLI). In Physlet's *Tracker* software, used to aid and automate point tracking in videos, the base and tip of each leaflet were tracked between initial stimulus and final equilibrium state. A protractor tool provided leaflet length and the protractor angle, exported at L2 and θ (Figure S1, Supporting Information).

Changes in L2 provided the angle about the rachis(θ_about_), and θ was the angle along the rachis (θ_along_). The quantitative model created to measure folding considers both as component factors of leaflet “folding”—dubbed the folding angle(θ_fold_). A fold angle of 0‐degree is the resting position of the leaflet, and a 90‐degree fold angle is fully folded. Graphics and calculations of this model can be seen in Figure 1B, Figure S4, and Equation S1 (Supporting Information).

## Conflict of Interest

The authors declare no conflict of interest.

## Supporting information

Supporting Information

## Data Availability

The data that support the findings of this study are available in the supplementary material of this article.
