# Supplementary material for: Plant Movement Response to Environmental Mechanical Stimulation Toward Understanding Predator Defense
Source: Adv Sci (Weinh). 2024 Aug 29;11(40):2404578. doi: 10.1002/advs.202404578 (PMC11516152; doi:10.1002/advs.202404578)

Supporting Information

Plant Movement Response to Environmental Mechanical Stimulation Toward Understanding Predator Defense

Alex Naglich^1^& Philip LeDuc^1,2,3,4,5*^

^1^Department of Mechanical Engineering, Carnegie Mellon University, Pittsburgh 15213, PA, USA

^2^Department of Biological Sciences, Carnegie Mellon University, Pittsburgh 15213, PA, USA

^3^Department of Biomedical Engineering, Carnegie Mellon University, Pittsburgh 15213, PA, USA

^4^Department of Computation Biology Engineering, Carnegie Mellon University, Pittsburgh 15213, PA, USA

^5^Department of Electrical and Computer Engineering, Carnegie Mellon University, Pittsburgh 15213, PA, USA

**Figure S1.** Point mass configuration in Physlet’s Tracker software, defining a protractor using two leaflet bases and the vertex and base and one leaflet tip as the arm.

**
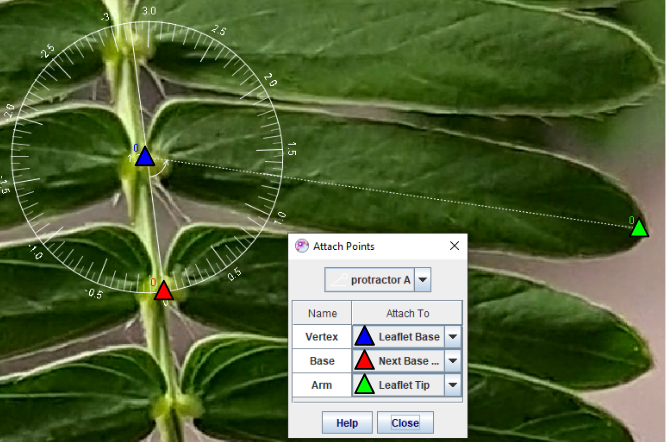
**

**Figure S2.** A. The poking mechanism assembled for construction and B. the poking mechanism in an exploded view. The Nema17 stepper motor(pink) would rotate the cam(dark blue), pushing on the ball bearing(green) and push the tip holder(orange) down, moving the blunt tip with constant velocity.

**
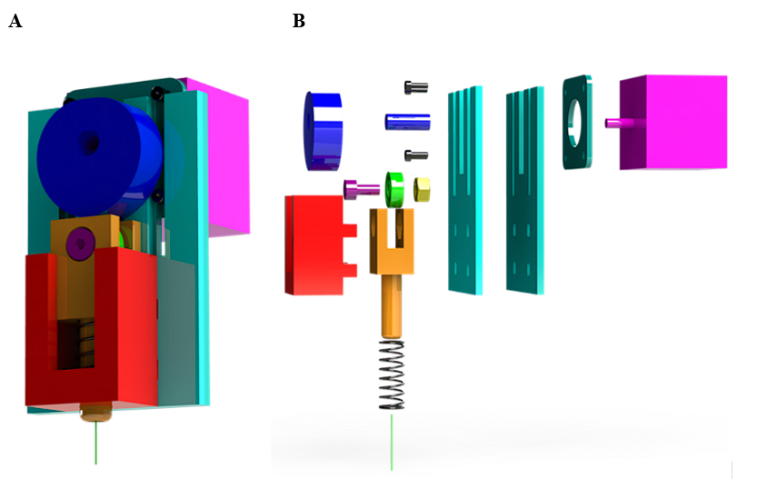
**.

**Figure S3**. Parametric equations driving the surface used in poking mechanism to prove linear velocity to poking motion. The line being driven by the equations is shown in black.


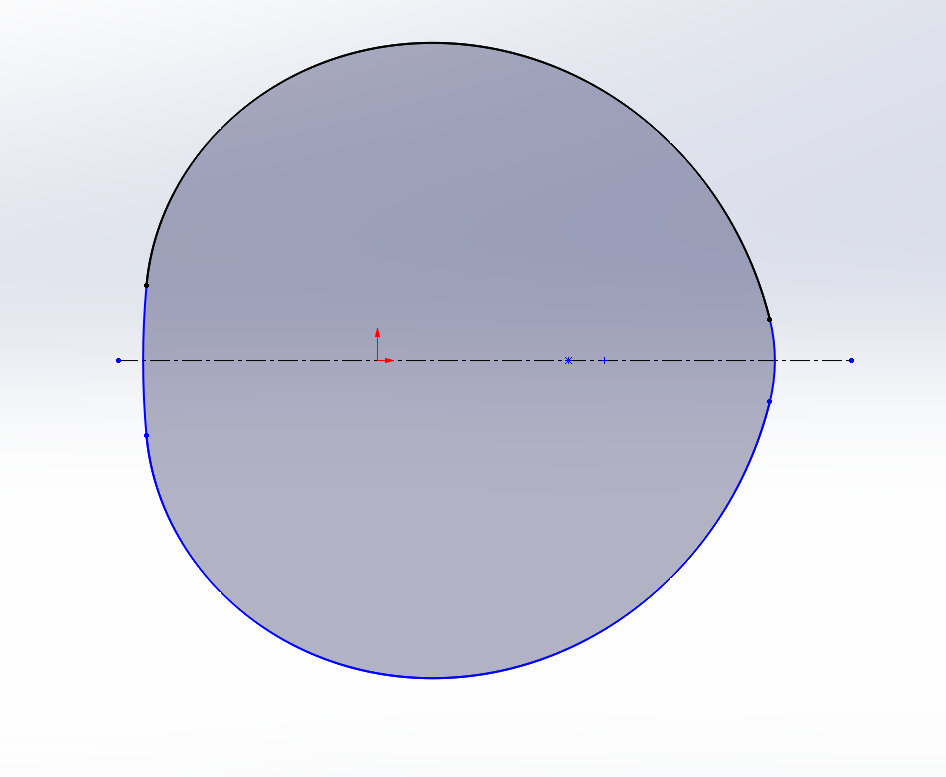

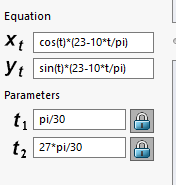


**Figure S4**. Graphical representation and derivation of Equation S1


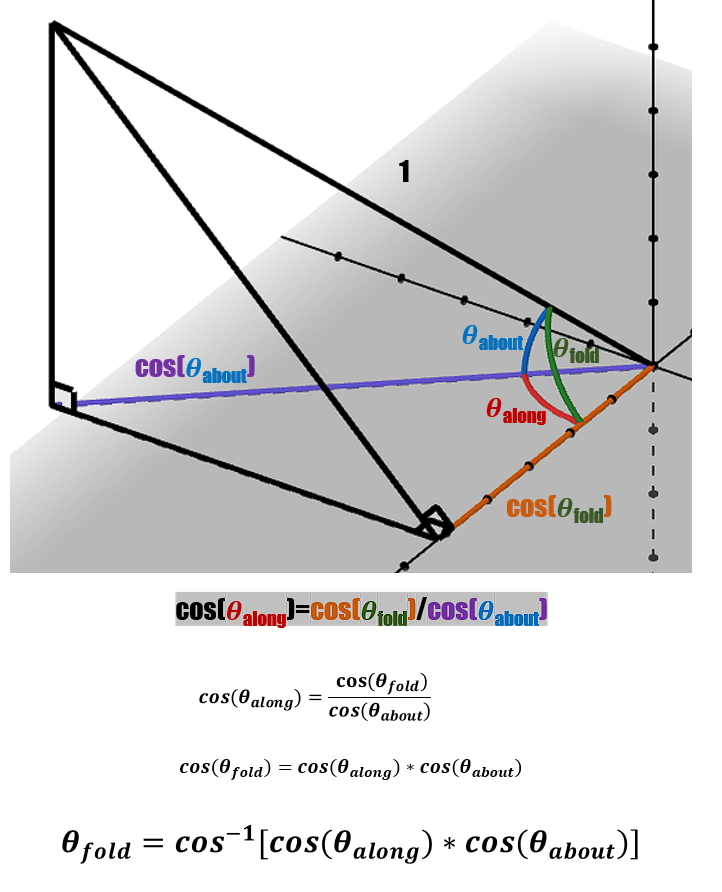


**Equation S1**: Equation relating folding angle to rotation both along and about the rachal axis.

$$\boldsymbol{\theta}_{\boldsymbol{fold}}\boldsymbol{=}{\boldsymbol{cos}^{\boldsymbol{-1}}\boldsymbol{[sin(\theta}}_{\boldsymbol{along}}\boldsymbol{)}\boldsymbol{*cos(\theta}_{\boldsymbol{about}}\boldsymbol{)]}$$

**Figure S5**. Example setup of scissors in position for removal of *M. pudica* and pinna immediately following removal*;* left: lateral view, right: top view.

**
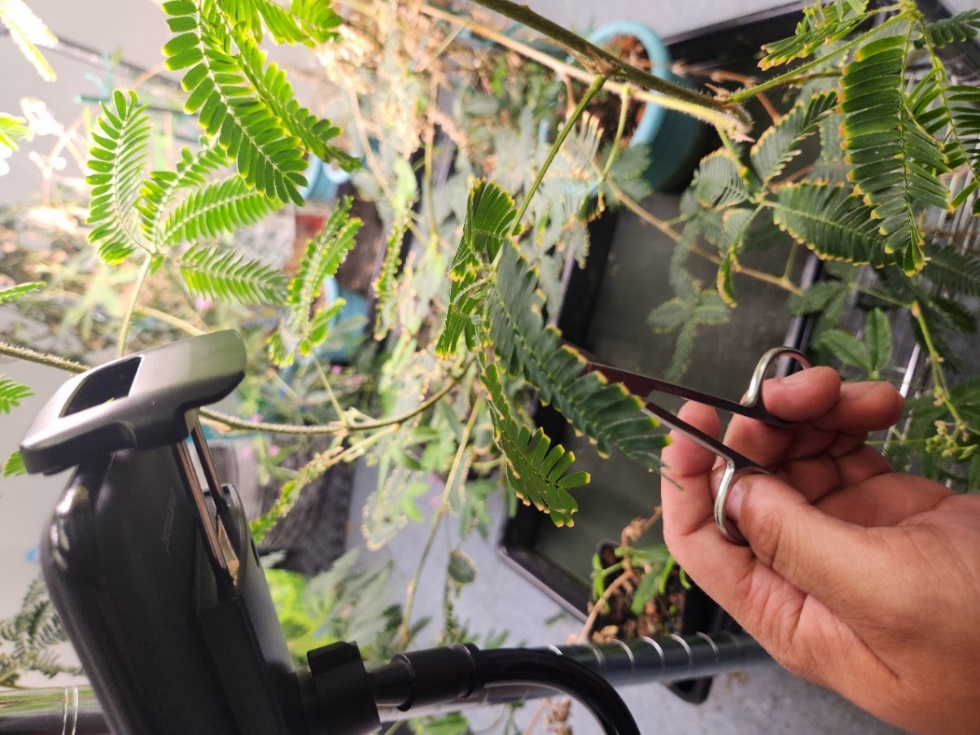
** **
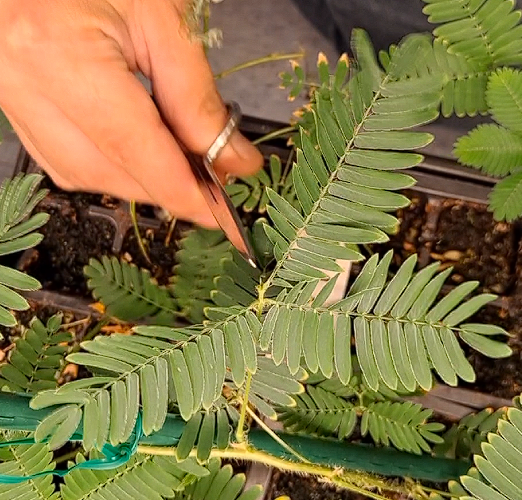
**

**Figure S6**. Example of poking mechanism in position for stimulation of *M. pudica;* left: lateral view, right: top view.


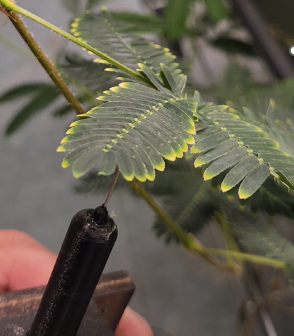

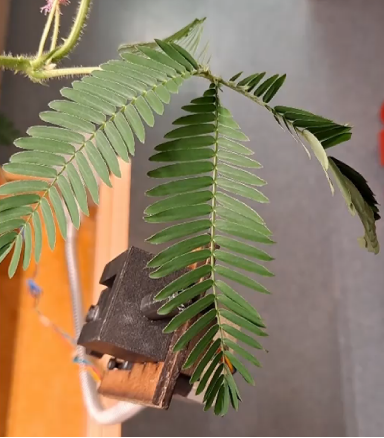


**Figure S7**. Example of Air Pulse Nozzle in position for stimulation of *M. pudica;* left: lateral view, right: top view.


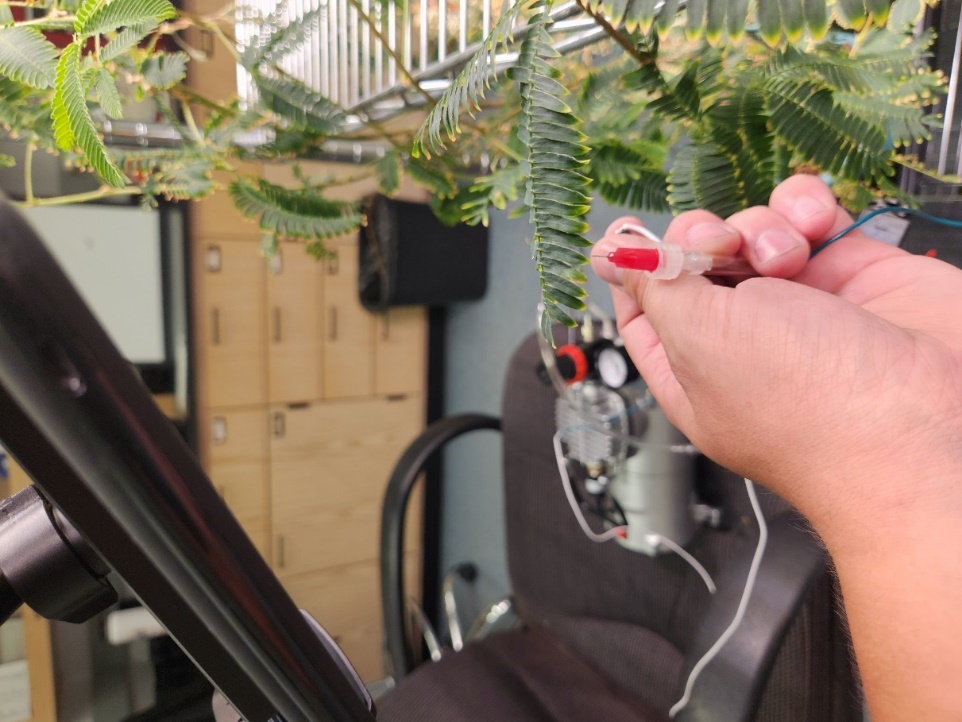

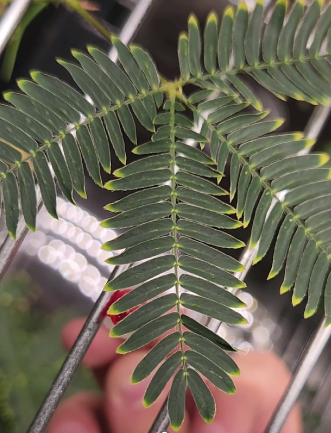

Supplement: Supplementary file 1 — Supporting Information [file ADVS-11-2404578-s001.docx]
